# 4-(Phenyl­sulfan­yl)benzene-1,2-dicarbonitrile

**DOI:** 10.1107/S1600536810043011

**Published:** 2010-10-30

**Authors:** Fei Yang, Fanjun Meng, Xiaomei Zhang

**Affiliations:** a Marine College, Shandong University at Weihai, Weihai 264209, People’s Republic of China; b School of Chemistry & Chemical Technology, Shandong University, Jinan 250100, People’s Republic of China

## Abstract

In the title compound, C_14_H_8_N_2_S, the dicyano-substituted aromatic ring and the phenyl ring attached to the central S atom adopt an angular V-shaped configuration. The dihedral angle between the rings is 103.6°.

## Related literature

The title compound is a precusor in the synthesis of phthalocyanine derivatives. For applications of phthalocyanines, see: Ao *et al.* (1995 ▶); Rey *et al.* (1998 ▶); Zhang *et al.* (2009 ▶); Beltrán *et al.* (2004 ▶); LukCentyanets (1999 ▶); Shirk & Pong (2000 ▶).
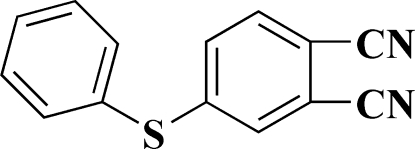


## Experimental

### 

#### Crystal data

C_14_H_8_N_2_S
*M* *_r_* = 236.28
Monoclinic, 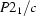

*a* = 7.8515 (7) Å
*b* = 9.7739 (9) Å
*c* = 15.6248 (14) Å
β = 91.544 (2)°
*V* = 1198.61 (19) Å^3^
*Z* = 4
Mo *K*α radiation
μ = 0.25 mm^−1^
*T* = 273 K
0.31 × 0.25 × 0.21 mm

#### Data collection

Bruker APEXII CCD diffractometer
Absorption correction: multi-scan (*SADABS*; Sheldrick, 2003 ▶) *T*_min_ = 0.928, *T*_max_ = 0.950
5758 measured reflections
2102 independent reflections
1818 reflections with *I* > 2σ(*I*)
*R*_int_ = 0.015

#### Refinement

*R*[*F* ^2^ > 2σ(*F* ^2^)] = 0.037
*wR*(*F* ^2^) = 0.098
*S* = 1.04
2102 reflections
154 parameters
17 restraints
H-atom parameters not refined
Δρ_max_ = 0.33 e Å^−3^
Δρ_min_ = −0.38 e Å^−3^

### 

Data collection: *APEX2* (Bruker, 2004 ▶); cell refinement: *SAINT-Plus* (Bruker, 2001 ▶); data reduction: *SAINT-Plus*; program(s) used to solve structure: *SHELXS97* (Sheldrick, 2008 ▶); program(s) used to refine structure: *SHELXL97* (Sheldrick, 2008 ▶); molecular graphics: *SHELXTL* (Sheldrick, 2008 ▶); software used to prepare material for publication: *SHELXTL*.

## Supplementary Material

Crystal structure: contains datablocks global, I. DOI: 10.1107/S1600536810043011/pb2042sup1.cif

Structure factors: contains datablocks I. DOI: 10.1107/S1600536810043011/pb2042Isup2.hkl

Additional supplementary materials:  crystallographic information; 3D view; checkCIF report

## Figures and Tables

**Fig. 1.**
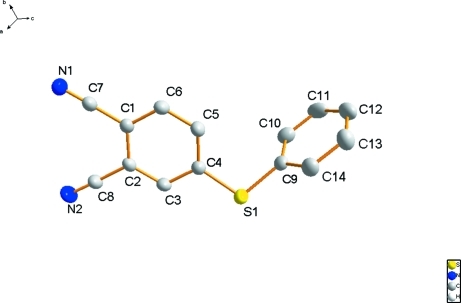
A view of (I) with the unique atom-labelling scheme. Displacement ellipsoids are drawn at the 30% probability level.

## supplementary crystallographic information

### Comment

Dicyano compounds have been widely used to synthesize many useful materials such as phthalocyanines. Phthalocyanines are an interesting class of compounds, with increasingly diverse industrial and biomedical applications, for instance as dyes and pigments, materials for optical storage (Ao *et al.* 1995), liquid crystals, oxidation catalysts, solar cell functional materials, gas sensors, nonlinear optical limiting devices (Shirk *et al.* 2000), photodynamic therapy agents (LukCentyanets *et al.* 1999), antimycotic material, and corrosion inhibitors (Zhang *et al.* 2009). The title compound 4-phenylsulfanylphthalonitrile was prepared according to the method reported in the literature.

The dicyano substituted phenyl ring and the aromatic ring attached to the sulfur atom is planar and the angle involving C4—S1—C9 (103.590) clearly indicate the angular orientation of the phenyl rings with respect to the sulfur atom with in this compound.

### Experimental

For general structure and background information on phthalocyanines, see: Zhang *et al.* (2009); For the synthesis, see: Rey *et al.* (1998).

### Refinement

Hydrogen atoms were placed in calculated positions and refined using a riding-model approximation with C—H = 0.93 Å, *U*_iso_ = 1.2*U*_eq_ (C) for aromatic H atoms and C—H = 0.96 Å, *U*_iso_ = 1.5*U*_eq_ (C) for methyl H atoms.

### Crystal data

**Table:**

C_14_H_8_N_2_S	*F*(000) = 488
*M**_r_* = 236.28	*D*_x_ = 1.309 Mg m^−^^3^
Monoclinic, *P*2_1_/*c*	Mo *K*α radiation, λ = 0.71073 Å
Hall symbol: -P 2ybc	Cell parameters from 2102 reflections
*a* = 7.8515 (7) Å	θ = 2.2–25.0°
*b* = 9.7739 (9) Å	µ = 0.25 mm^−^^1^
*c* = 15.6248 (14) Å	*T* = 273 K
β = 91.544 (2)°	Block, colorless
*V* = 1198.61 (19) Å^3^	0.31 × 0.25 × 0.21 mm
*Z* = 4	

### Data collection

**Table:**

Bruker APEXII CCD diffractometer	2102 independent reflections
Radiation source: fine-focus sealed tube	1818 reflections with *I* > 2σ(*I*)
graphite	*R*_int_ = 0.015
phi and ω scans	θ_max_ = 25.0°, θ_min_ = 2.5°
Absorption correction: multi-scan (*SADABS*; Sheldrick, 2003)	*h* = −8→9
*T*_min_ = 0.928, *T*_max_ = 0.950	*k* = −11→11
5758 measured reflections	*l* = −14→18

### Refinement

**Table:**

Refinement on *F*^2^	Primary atom site location: structure-invariant direct methods
Least-squares matrix: full	Secondary atom site location: difference Fourier map
*R*[*F*^2^ > 2σ(*F*^2^)] = 0.037	Hydrogen site location: inferred from neighbouring sites
*wR*(*F*^2^) = 0.098	H-atom parameters not refined
*S* = 1.04	*w* = 1/[σ^2^(*F*_o_^2^) + (0.043*P*)^2^ + 0.3751*P*] where *P* = (*F*_o_^2^ + 2*F*_c_^2^)/3
2102 reflections	(Δ/σ)_max_ < 0.001
154 parameters	Δρ_max_ = 0.33 e Å^−^^3^
17 restraints	Δρ_min_ = −0.38 e Å^−^^3^

### Special details

**Table:**

Geometry. All e.s.d.'s (except the e.s.d. in the dihedral angle between two l.s. planes) are estimated using the full covariance matrix. The cell e.s.d.'s are taken into account individually in the estimation of e.s.d.'s in distances, angles and torsion angles; correlations between e.s.d.'s in cell parameters are only used when they are defined by crystal symmetry. An approximate (isotropic) treatment of cell e.s.d.'s is used for estimating e.s.d.'s involving l.s. planes.
Refinement. Refinement of *F*^2^ against ALL reflections. The weighted *R*-factor *wR* and goodness of fit *S* are based on *F*^2^, conventional *R*-factors *R* are based on *F*, with *F* set to zero for negative *F*^2^. The threshold expression of *F*^2^ > σ(*F*^2^) is used only for calculating *R*-factors(gt) *etc*. and is not relevant to the choice of reflections for refinement. *R*-factors based on *F*^2^ are statistically about twice as large as those based on *F*, and *R*- factors based on ALL data will be even larger.

### Fractional atomic coordinates and isotropic or equivalent isotropic displacement parameters (Å^2^)

**Table:**

	*x*	*y*	*z*	*U*_iso_*/*U*_eq_	
S1	1.02062 (6)	−0.13035 (5)	0.20832 (4)	0.0706 (2)	
N1	1.1713 (2)	0.44401 (17)	−0.06030 (11)	0.0689 (5)	
N2	1.5357 (2)	0.1851 (2)	−0.00461 (12)	0.0759 (5)	
C1	1.10678 (19)	0.23614 (16)	0.03502 (9)	0.0428 (4)	
C2	1.23816 (19)	0.14420 (16)	0.05715 (10)	0.0437 (4)	
C3	1.2075 (2)	0.03475 (17)	0.11030 (11)	0.0496 (4)	
H3	1.2954	−0.0253	0.1252	0.060*	
C4	1.0448 (2)	0.01386 (16)	0.14187 (11)	0.0466 (4)	
C5	0.9148 (2)	0.10349 (18)	0.11842 (11)	0.0508 (4)	
H5	0.8056	0.0890	0.1383	0.061*	
C6	0.9453 (2)	0.21380 (17)	0.06595 (11)	0.0504 (4)	
H6	0.8571	0.2735	0.0512	0.061*	
C7	1.1412 (2)	0.35200 (18)	−0.01832 (11)	0.0498 (4)	
C8	1.4050 (2)	0.16575 (18)	0.02351 (12)	0.0539 (4)	
C9	0.8157 (2)	−0.10711 (17)	0.25164 (11)	0.0493 (4)	
C10	0.7880 (3)	−0.0083 (2)	0.31328 (12)	0.0629 (5)	
H10	0.8762	0.0491	0.3316	0.075*	
C11	0.6272 (3)	0.0044 (2)	0.34748 (13)	0.0723 (6)	
H11	0.6075	0.0708	0.3887	0.087*	
C12	0.4977 (3)	−0.0807 (2)	0.32069 (14)	0.0724 (6)	
H12	0.3900	−0.0715	0.3434	0.087*	
C13	0.5263 (3)	−0.1780 (2)	0.26123 (15)	0.0736 (6)	
H13	0.4380	−0.2358	0.2437	0.088*	
C14	0.6849 (2)	−0.1927 (2)	0.22626 (12)	0.0601 (5)	
H14	0.7032	−0.2604	0.1857	0.072*	

### Atomic displacement parameters (Å^2^)

**Table:**

	*U*^11^	*U*^22^	*U*^33^	*U*^12^	*U*^13^	*U*^23^
S1	0.0552 (3)	0.0589 (3)	0.0988 (4)	0.0124 (2)	0.0230 (3)	0.0307 (3)
N1	0.0704 (11)	0.0639 (10)	0.0736 (11)	0.0084 (8)	0.0214 (8)	0.0192 (9)
N2	0.0501 (10)	0.0816 (12)	0.0971 (13)	0.0050 (8)	0.0241 (9)	0.0081 (10)
C1	0.0433 (8)	0.0425 (8)	0.0428 (8)	0.0005 (7)	0.0046 (6)	−0.0009 (7)
C2	0.0387 (8)	0.0459 (9)	0.0470 (9)	0.0015 (7)	0.0079 (6)	−0.0033 (7)
C3	0.0418 (9)	0.0483 (9)	0.0590 (10)	0.0091 (7)	0.0062 (7)	0.0042 (8)
C4	0.0442 (9)	0.0439 (9)	0.0520 (9)	0.0004 (7)	0.0064 (7)	0.0012 (7)
C5	0.0373 (8)	0.0530 (10)	0.0623 (10)	0.0005 (7)	0.0082 (7)	0.0071 (8)
C6	0.0397 (8)	0.0517 (9)	0.0600 (10)	0.0069 (7)	0.0039 (7)	0.0072 (8)
C7	0.0458 (9)	0.0522 (10)	0.0519 (9)	0.0062 (8)	0.0100 (7)	0.0016 (8)
C8	0.0449 (9)	0.0535 (10)	0.0638 (11)	0.0065 (8)	0.0098 (8)	0.0049 (8)
C9	0.0517 (9)	0.0456 (9)	0.0510 (9)	0.0027 (7)	0.0070 (7)	0.0115 (7)
C10	0.0732 (12)	0.0524 (10)	0.0626 (11)	−0.0007 (9)	−0.0059 (9)	0.0000 (9)
C11	0.0989 (16)	0.0647 (12)	0.0539 (11)	0.0253 (12)	0.0141 (11)	−0.0029 (9)
C12	0.0647 (12)	0.0775 (14)	0.0762 (14)	0.0134 (11)	0.0239 (10)	0.0126 (11)
C13	0.0578 (11)	0.0763 (14)	0.0875 (15)	−0.0109 (10)	0.0137 (10)	−0.0022 (12)
C14	0.0647 (11)	0.0573 (11)	0.0588 (11)	−0.0039 (9)	0.0129 (9)	−0.0059 (9)

### Geometric parameters (Å, °)

**Table:**

S1—C4	1.7638 (16)	C5—H5	0.9300
S1—C9	1.7770 (17)	C6—H6	0.9300
N1—C7	1.142 (2)	C9—C14	1.375 (3)
N2—C8	1.143 (2)	C9—C10	1.385 (3)
C1—C6	1.386 (2)	C10—C11	1.389 (3)
C1—C2	1.404 (2)	C10—H10	0.9300
C1—C7	1.436 (2)	C11—C12	1.370 (3)
C2—C3	1.379 (2)	C11—H11	0.9300
C2—C8	1.440 (2)	C12—C13	1.353 (3)
C3—C4	1.397 (2)	C12—H12	0.9300
C3—H3	0.9300	C13—C14	1.381 (3)
C4—C5	1.387 (2)	C13—H13	0.9300
C5—C6	1.379 (2)	C14—H14	0.9300
			
C4—S1—C9	103.59 (7)	N2—C8—C2	178.3 (2)
C6—C1—C2	119.07 (14)	C14—C9—C10	119.68 (17)
C6—C1—C7	120.97 (14)	C14—C9—S1	119.29 (14)
C2—C1—C7	119.95 (14)	C10—C9—S1	120.97 (14)
C3—C2—C1	120.38 (14)	C9—C10—C11	119.38 (18)
C3—C2—C8	120.55 (14)	C9—C10—H10	120.3
C1—C2—C8	119.07 (14)	C11—C10—H10	120.3
C2—C3—C4	120.13 (14)	C12—C11—C10	120.21 (18)
C2—C3—H3	119.9	C12—C11—H11	119.9
C4—C3—H3	119.9	C10—C11—H11	119.9
C5—C4—C3	119.22 (15)	C13—C12—C11	120.05 (19)
C5—C4—S1	124.76 (12)	C13—C12—H12	120.0
C3—C4—S1	116.01 (12)	C11—C12—H12	120.0
C6—C5—C4	120.80 (15)	C12—C13—C14	120.9 (2)
C6—C5—H5	119.6	C12—C13—H13	119.6
C4—C5—H5	119.6	C14—C13—H13	119.6
C5—C6—C1	120.39 (15)	C9—C14—C13	119.80 (18)
C5—C6—H6	119.8	C9—C14—H14	120.1
C1—C6—H6	119.8	C13—C14—H14	120.1
N1—C7—C1	178.87 (19)		
